# Voices of children with intellectual disabilities on participation in daily activities

**DOI:** 10.4102/ajod.v10i0.792

**Published:** 2021-07-05

**Authors:** Karina Huus, Refilwe Morwane, Maria Ramaahlo, Sadna Balton, Emelie Pettersson, Ingalill Gimbler Berglund, Shakila Dada

**Affiliations:** 1 Department of Nursing, School of Health and Welfare, Jönköping University, Jönköping, Sweden; 2 CHILD Research Group, Jönköping University, Jönköping, Sweden; 3 Swedish Institute for Disability Research (SIDR), Jönköping, Sweden; 4 Centre for Augmentative and Alternative Communication, University of Pretoria, Pretoria, South Africa; 5 Department of Speech Therapy and Audiology, Chris Hani Baragwanath Academic Hospital, Soweto, South Africa

**Keywords:** intellectual disabilities, participation, Picture my Participation, self-ratings, children, barriers, facilitators, daily life

## Abstract

**Background:**

Participation in daily activities is expressed as a human right. Full participation of children with disabilities in daily activities creates optimal opportunities for learning and development. Previous studies have focused primarily on proxy ratings of participation of children with intellectual disabilities in daily activities. However, little is known about how the children rate barriers and facilitators to their participation in everyday activities.

**Objectives:**

To identify barriers to and facilitators for everyday activities as experienced by children with intellectual disabilities from low- and middle-income countries and high-income countries. The research questions were as follows: ‘what barriers to participation do children with disabilities experience in everyday activities?’ and ‘what facilitators to participation do children with disabilities experience in everyday activities?’

**Method:**

A qualitative content analyses was used in this study, and individual interviews were conducted with 49 children with intellectual disabilities. The interviews were performed using pictures. The children also selected the most important activities and described in their own words the facilitators and barriers relevant to being able to perform the activities.

**Results:**

The most important activities were organised leisure activities, formal learning at school, taking care of other family members and family mealtimes. Self-reported barriers identified were personal functioning, social exclusion and lack of resources. The identified facilitators included satisfaction, personal capability, being included and having access to resources.

**Conclusion:**

These findings provide important knowledge about the factors to consider in the development of interventions, aimed at improving the participation of children with intellectual disabilities.

## Introduction

Participation within the environment has been identified as an important health outcome for both children with disabilities and their families (Arvidsson et al. 2014; Coster & Khetani 2008; Imms et al. 2017) However, research studies have indicated that children with disabilities are restricted in their participation in daily activities, such as sports, educational opportunities, and recreational and informal leisure activities (Conchar et al. 2016; Higashida 2017; Moyi 2017). In a recent review that focused on children with disabilities from low- and middle-income countries (LMICs), it was found that children with disabilities participate less in everyday activities and have fewer engagements than their non-disabled peers (Schlebusch et al. 2020). More specifically, children with intellectual disabilities experience restriction in their participation (Hansen, Siame & Van der Veen 2014) because of issues, such as stigma and discrimination (Ferguson 2014). Whilst there is an urgent need to develop interventions that promote participation, there is limited information available regarding facilitators that enable participation of children with intellectual disabilities. Studies that focus on the barriers and facilitators to participation of children with disabilities tend to focus on participation in physical activities only (Alesi & Pepi 2017; Maciver et al. 2019; Shields & Synnot 2016), and these are sometimes limited to high-income countries (McKenzie, McConkey & Adnams 2013).

Shields, Synott and Barr (2012) conducted a systematic review of the perceived barriers and facilitators to physical activity in children with disabilities, as perceived by children, parents and health professionals (organisational staff). Of the 14 studies reviewed, only five studies included children with disabilities, whilst one study included both parents and children as participants, with the other studies being proxy ratings. A range of personal, social, environmental and policy or programme-related barriers and facilitators were identified. Overall, the perceived barriers and facilitators identified by the different groups of participants were similar, but the emphasis placed on the different themes varied between groups. Children with disabilities most commonly identified personal, social and environmental barriers to participation (Shields et al. 2012), whilst parents focused predominantly on social, policy and programme barriers or on their own involvement in their child’s activity. An important finding of the Shields et al.’s (2012) study was that the parents identified more facilitators than the children, as all studies in the review often neglected to ask children about facilitators to their physical activity. The health professionals, on the other hand, identified barriers and facilitators related to policies and programmes (Shields et al. 2012).

It is evident from Shields et al. (2012) that there is a paucity of literature regarding the voices of children with disabilities and their perceptions of their participation in activities. Moreover, research in the field of disabilities pertaining to facilitators and barriers to participation of children in daily activities is either specifically on the views of youths with disabilities aged 7–17 years (King et al. 2013) or on proxy ratings (i.e. caregiver responses on behalf of children with disabilities) (Conchar et al. 2016; Imms et al. 2016; Moyi 2017; Wright et al. 2019). In a study by Huus et al. (2015), self-ratings of children with intellectual disabilities did not always overlap with primary caregiver proxy ratings on the realisation of children’s rights. Interestingly, differences in self- and proxy ratings were found to be affected by the complexity of the children’s needs in Maslow’s hierarchy, with caregivers more often giving the same answer as the children when the needs of the children were at the lower end of Maslow’s hierarchy. When the questions related to more complex needs such as having someone to play with, the agreement between primary caregivers and the children was less likely to overlap. Basic needs for survival are easier to fulfil and objectively see, but when it comes to more complex needs for participation in daily activities it is very difficult for primary caregivers to realise the needs of the child. Therefore, the agreement between the proxy ratings of children and their primary caregivers depends on the complexity of the Maslow hierarchy, and children’s own voices are even more important (Huus et al. 2015).

Article 12 of the United Nations Convention on the Rights of the Child states that children have the right to express their views freely in matters affecting them (United Nations General Assembly 1989). Studies that have explored barriers and facilitators to participation as experienced by children with disabilities seldom include the voices of children with disabilities (Adair et al. 2015; Åström, Khetani & Axelsson 2018). Barriers are defined as factors that hinder the participation of children in everyday life situation. Facilitators, however, include strategies that can facilitate their participation. Children with intellectual disabilities experience social exclusion on a much greater scale than their non-disabled peers. Mckenzie et al. (2013) argued that this experience is intensified within contexts of LMICs. Research on the perceptions of persons with intellectual disabilities and their families is predominantly conducted in and about high-income countries (McKenzie et al. 2013)

Participation has been explained by the World Health Organization’s (WHO) International Classification of Functioning, Disability (ICF) (WHO 2018) and the Convention on the Rights of the Child (United Nations General Assembly 1989) as the involvement in life situations, that is for example being listened to and having the right to education and a family. In the ICF that uses the WHO’s definition of disability, an individual’s health condition is intertwined and in constant interaction with the environment, in which they function. Domains described in the ICF encompass an individual’s impairment (i.e. body function and structure), activity limitation (i.e. difficulty experienced by persons in executing a task), as well as participation restrictions (i.e. challenges experienced by an individual’s involvement in life situations) (WHO 2018). This definition suggests not only that disability is attributed to the person’s health condition but also that other factors extrinsic to the individual, such as the environment in which they live, can play an important role. The ICF, therefore, follows a biopsychosocial approach to disability.

The WHO further defines participation as involvement in a life situation (WHO 2018). However, the definition of participation has not been consistent. Imms et al. (2017) described the language and construct of participation as consisting of two dimensions, namely, attendance and engagement. Attendance refers to ‘being there’, whilst engagement refers to involvement in an activity. The Family of Participation-Related Constructs also describes processes within the person that influence participation. These processes include preferences, sense of self and activity competencies. Preferences are activities that the child finds as meaningful or that are interesting, and sense of self involves satisfaction with the activities, confidence in themselves and self-esteem. Activity competence is related to the ability to perform in the activity in a manner that is expected; this includes both cognitive and physical abilities (Imms et al. 2017). Legislation frameworks, such as the Convention on the Rights of Persons with Disabilities and Conventions on the Rights of Children, emphasise the importance of attendance, that is, being there, engagement in activities is not emphasised and is often overlooked (Granlund 2013). The ICF as a framework focusses on the objective aspect of participation (Ueda & Okawa 2003). However, few studies have considered subjective aspects of participation for children with disabilities, particularly children with intellectual disabilities (Arvidsson et al. 2014; Coster & Khetani 2008).

Despite the importance of self-ratings, there are disadvantages that arise from asking children with intellectual disabilities to self-report. These disadvantages include the potential difficulty in comprehending and responding to questions and difficulty in recalling the past events related to participation in activities. It is important to note that the proxy does not have the same perspective as the child (Jokovic, Locker & Guyatt 2004; Nilsson et al. 2015). It is, therefore, plausible that caregivers’ knowledge of their children is limited, particularly with respect to relationships and activities that occur outside the home and with respect to internal feeling states. Thus, researchers should consider instruments that allow children with disabilities to share their experiences and perspectives about the barriers and facilitators to their participation (Huus et al. 2015; Nilsson et al. 2015). The study aims to identify barriers and facilitators for everyday activities as experienced and reported by children with intellectual disabilities from South Africa and Sweden. This aim leads to the research questions: ‘what barriers to participation do children with disabilities experience in everyday activities?’ and ‘what facilitators to participation do children with disabilities experience in everyday activities?’

## Research methods and design

A qualitative research design was used in this study. Individual interviews were conducted with 49 children with intellectual disabilities, in which they rated their participation in activities of daily living using a self-reporting measurement instrument, namely, Picture my Participation (PMP). The psychometric properties are described in Arvidsson et al. (2019). The interviews were performed as a conversation with an interview guide using pictures. The children also selected the most important activities and described in their own words the facilitators and barriers relevant to being able to perform the activities. Inductive content analysis was used.

The use of interviews enables the researcher to obtain first-hand perspectives, views, experiences and beliefs and/or motivations of individuals on a specific topic (Creswell 2014). The use of the open-ended interview questions in the PMP in this study, allowed the researchers a ‘deeper’ understanding of the phenomena than would be obtained from purely quantitative methods, such as questionnaires with set answers.

This study formed part of a larger collaborative project between South Africa and Sweden, with data collected from both the countries focussed on the participation of children with intellectual disabilities in everyday activities. South Africa is an upper-middle-income country with various challenges regarding the access to healthcare and education for children with disabilities (Moodley & Ross 2015). Sweden, however, is a high-income country that not only is well resourced but also faces challenges in improving the participation of children with disabilities in daily activities. The larger collaboration project aimed at exploring the usefulness of the PMP in different countries, such as LMICs (South Africa) and high-income country (Sweden). Arvidsson et al. (2019) found the instrument useful in both countries for children with intellectual disabilities.

The focus of this study was on the broader understanding of the barriers and facilitators to participation in daily activities, as reported by children with intellectual disabilities broadly. Therefore, comparisons between the countries were not the focus of the study, as previous studies have found similarities in contexts on the PMP measure.

### Ethical considerations

Ethical approval to conduct this study was obtained from the ethics committee of a University of Pretoria in South Africa, as well as the relevant department of education, and from the research ethics committee of Linköping University in Sweden with reference numbers: GW20180301HS and Dnr 2016/544-31. Caregivers of the participants gave consent for their children to participate in the study, and assent was obtained from the children. Participants were required to be competent to converse in English or Swedish (determined by self-report with additional input from the teachers of the children). Schools for children with intellectual disabilities in South Africa and Sweden were selected to be included in the study.

### Participants

A non-probability, purposive sampling method was used in this study. Out of 49 children, 31 children with intellectual disabilities from South Africa (SA) and 18 children from Sweden (SWE) formed part of the study. Schools for children with special needs were contacted, and the teachers distributed information about the study to the parents and those parents who wanted to participate in the study sent their consent to the researchers. The schools were located in different parts of Sweden and South Africa. The ages of children ranged from 7 to 18 years. The children were all diagnosed with intellectual disability. Caregivers described their child’s level of functioning by using the Ten Questions Questionnaire (TQQ) (Mung’ala-Odera et al. 2004). The children were, therefore, identified as having mild (42.60%: SA; 62.5%: SWE), moderate (45.90%: SA; 31.30%: SWE) and severe (11.50%: SA; 6.30%: SWE) intellectual impairment by their caregivers.

### Instruments

The TQQ was completed by caregivers prior to obtaining self-ratings regarding participation from the children. The questionnaire was used to screen for severe neurological impairment in children from low-resourced countries and had also been successfully used in high-income countries. The questionnaire comprised 10 questions related to motor, speech and language, cognition, as well as health condition (Durkin et al. 1994). This instrument was used to decide on the child’s functioning.

The main instrument used in this study was PMP, which is designed to capture the two participation dimensions of attendance and involvement of children and youth with mild intellectual disability who live in low-resource settings (Arvidsson et al. 2019; Dada et al. 2020). Picture my Participation measures participation in 20 home, social and community activities, and is performed as part of a structured interview with children. The open-ended questions where the children described barriers and facilitators for participation with their own words were transcribed and analysed by qualitative content analysis in this study. The items of PMP were selected by reviewing the existing participation measures and matching items to the UNCRC. The content of the 20 items were found to be valid in the LMIC context (South Africa) and for children with intellectual disability (Arvidsson et al. 2019).

Picture my Participation is a manual-based structured interview instrument. It comprises three trial items where the ability of the child to understand the concepts’ frequency of attending and engagement with the help of graphic symbols and the scale anchors illustrated by graphic symbols is tested. After the trial items, four sections follow with the purpose of (1) determining perceived attendance in various activities using a four-point Likert-type scale, (2) determining perceived involvement in various activities using a four-point Likert-type scale, (3) prioritising activities considered to be the three most important to the child and (4) determining perceived barriers and facilitators to participation. The last two purposes are the focus of this current study.

### Data collection

Data were collected by the authors and two other researchers in the research group who all are experienced in interviewing children and familiar with the PMP instrument conducted interviews with the children with intellectual disabilities. The researchers sent TQQ forms via the school to the caregivers to complete and obtain consent for their children to participate in the study. After consent was obtained, assent from the children was obtained. The interviews were conducted with the children individually using the PMP instrument. In order to facilitate the communication with the children, a picture support approach, named the Talking Mats^TM^ (Cameron & Murphy 2002), was used. The pictures used in the interviews were graphical symbols from the Picture Communication Symbols (PCS^TM^) (Fuller & Lloyd 1997). Each child’s individual interview lasted for approximately 20–30 min and was conducted on the school premises. The children were required to identify three items that were most important for them from the 20 items on the PMP.

Thereafter, the children were asked to identify barriers and facilitators to participating in those three specified activities. The children were asked, ‘is there anything that makes it difficult for you to participate in the activity’. If they said ‘yes’, they were asked, ‘what is it that makes it difficult for you to participate?’ Likewise, the children were asked, ‘is there anything that makes it easier to participate in the activity?’ and ‘what is it that makes it easier for you to participate?’. The children responded verbally, and their answers were transcribed verbatim by the researchers. Because of the children’s limited language development, the answers were short and comprised a few words or a sentence.

### Data analysis

An inductive qualitative content analysis was used to analyse the transcribed data on identified barriers and facilitators (Jay et al. 1983). The researchers employed the technique referred to as ‘conventional qualitative content analysis’, in which categories are created directly and inductively from the collected data (Hsieh & Shannon 2005). This method of analysis consists of an inductive, reflexive analysis that focuses on the emergence of ideas, codes and thematic structures (Soffer & Chew 2015). Member checking was only conducted during the interview to ensure that the interviewer had correctly understood the message the participant was intending to convey. Furthermore, member checking of the coded transcripts, although desirable, was not done.

Inductive coding was performed. In order for the voice of the children to be heard, the analysis was performed close to the text. Coding was conducted by two authors independently, and where there were discrepancies, recoding of the data was not carried out until consensus was reached. This peer review of coding enhanced the interpretive rigour of the analysis (Elo & Kyngäs 2008). In the results presented below, citations from the children are presented in quotes.

## Results

The children were asked to choose the three most important activities for participation from a total of 20. However, some of the children chose one or two activities as the most important to them rather than three as instructed. Nonetheless, the majority of the children provided a choice of three most important activities.

Activities that the children reported most frequently as **most** important to them were organised leisure activities, formal learning at school, taking care of other family members and taking part in family mealtimes. The activities **least** mentioned as important to them include gathering daily necessities for the family, family or community celebrations, taking part in social activities in the community and getting together with other children in the community (Table 1).

**TABLE 1:** The activities listed according to the number of children identifying them as most important and those activities least mentioned as important.

Most frequently mentioned as most important activities	Least frequently mentioned as most important activities
Organised leisure activities (*n* = 15)	Gathering daily necessities for the family (*n* = 2)
Formal learning at school (*n* = 14)	Family/community celebrations (*n* = 2)
Taking care of other family members (*n* = 13)	Taking part in social activities in the community (*n* = 2)
Family mealtimes (*n* = 11)	Getting together with other children in the community (*n* = 3)

### Perceived barriers and facilitators to participation in daily activities

From the inductive content analysis, three categories for barriers and four categories for facilitators emerged, as shown in Table 2. The data from both Sweden and South Africa were presented together, and no comparisons between the two countries were made because the results were comparable in both settings. There was also a disparity in the numbers of participants from the two countries.

**TABLE 2:** Categories for barriers and facilitators.

Barriers	Facilitators
Personal functioning	Satisfaction
Social exclusion	Personal capability
Lack of resources	Being included
-	Having resources

### Barriers: Description of the categories

The analysis of children’s perceptions of barriers to participation in daily activities resulted in three categories: *Personal functioning, social exclusion and lack of resources.*

#### Personal functioning

Personal functioning was described by the children as factors within the person, such as their view of the self in relation to their physical or psychological functioning

One of the barriers was the lack of knowledge regarding how to take care of their own health, ‘to see what is wrong with you’. Barriers identified by the children were, therefore, about fear of healthcare procedures and healthcare professionals, as evident in the following quote: ‘I do not trust the dentist; I am scared of the dentist’.

Another barrier identified included the children’s view of their self, where they felt an inability to perform activities on their own, for example, in relation to meal preparation for the family, which is clear in the below quote: ‘when I do it myself it’s very hard’. Quiet leisure activities, such as storybook reading, could also be considered as a barrier when the children did not have the ability to read the books they were provided with.

The children described the school as boring and certain subjects were described as barriers because they were perceived as difficult for them, such as ‘Afrikaans and math’. In addition, there was a barrier related to the policy enforcing activities in school for the children. The children said that they felt they were made to do things they did not want to do in the school, for example, ‘to have to go outside when it is wet’, and they were also overloaded with instructions and there were too many disturbances.

The children did not like being out of their ‘comfort zone’. An example of this was when a child was not comfortable with being on stage during the church service. Further challenges were also reported when caring for pets, and the children perceived that they had difficulty in preventing the pet from harming others. Finally, other barriers reported were related to restricted participation in organised leisure activities, for example, ‘not being able to play due to injury’. This was mainly because of the children’s perceived lack of body function.

#### Social exclusion

Social exclusion was described as a barrier in relation to others, such as the children’s interaction with other people, being excluded from the social group and not being accepted on equal terms in these groups. The children felt ignored as they stated that ‘some people don’t listen’. The children also described a barrier when they talked about being outside of the social fellowship. They were excluded from being in the company of others when in a social activity or when they did not have friends to play with, as evident in the following quote: ‘there are not so many children outside and they do not answer when I call’.

The children also described social exclusion as a barrier when in their own homes or with families. It could be difficult for the children to take care of other family members because they did not see the others, or they had difficulty making their voice heard. They also described social exclusion as a barrier at mealtimes when there was poor communication within the family, ‘mum, grandmother, brother don’t communicate much’) or when they were not allowed to be part of other activities in the family and were left by themselves.

A barrier to participation in formal learning at school was social exclusion, which was described by the children as being bullied by peers and teachers at school in different ways. It could include being called names by their peers or not making friends, as shown in the quote ‘when friends call me names and backchat’. However, it could also come from the teachers, singling out the child with a disability, as the below quote mentions, ‘when we go to other teachers and they shout it makes my ears sore and they blame me for everything’. Furthermore, they feared being physically harmed when engaging in contact-sports activities: ‘when they kick you when you have the ball’.

#### Lack of resources

Lack of resources refers to economic constraints to participation, such as lack of money. An example provided was related to not having money to run errands and shopping, ‘not having money to buy’. And not having resources to buy necessary items became a barrier within the school setting: ‘you wear your own clothes, not a uniform. You can’t when you cannot pay’.

It could also refer to the difficulties with transportation or lack of resources with transportation, for example, going to church, ‘driving a long way’, ‘to walk there’.

### Facilitators: Description of the categories

The analysis of children’s perceptions of facilitators for participation in daily activities resulted in four categories: *satisfaction, personal capability, being included* and *having resources*.

#### Satisfaction

The children described how participation in activities was facilitated by pleasure, and, therefore, the satisfaction they gained from doing the activity, and they said that they enjoyed what they were doing. Participating in cleaning the house and organised leisure activities enhanced the pleasure of the children, ‘have fun and enjoy myself’. The children described satisfaction as a facilitator in taking care of other family members, ‘easy because I help and love my family’ and to have satisfaction in religion without being shy about it and to trust in God, ‘pray to God and he will answer’.

Satisfaction was also described as having a common interest with someone else, ‘music be out and play’ and ‘we sing’. Family mealtimes were a source of satisfaction, when the child enjoyed cooking. Quiet leisure activities were also described as satisfactory activities, ‘to read in the morning and evening’ and ‘being alone’.

Personal care was facilitated when the children felt satisfaction with the chores in daily routines for personal care and looking after their own health, ‘feel like doing it’. A source of satisfaction that facilitated personal care was the sense of wanting to be of assistance, ‘likes to help mum’.

#### Personal capability

A view of self as having capability was described in terms of social and physical skills, ‘good interaction skills’ and ‘you can play and score’.

Attendin*g* school was also perceived as a facilitator of learning, ‘makes me concentrate and calm’. Perceived personal capability was a facilitator when taking care of other family members and family pets. In order to have the ability to look after someone else could be described as ‘just easy’ and ‘being able to look after family members’.

Personal capability could also be to have mental and not just physical capability in interaction with other people in social activities, ‘I trust them, I am trying to gather courage. It’s important to do that, you cannot just skip it’. To feel responsibility and personal capability towards family was a facilitator to participate in daily life, ‘I will never leave let them down’. Included in personal capability was the children describing how they behaved at mealtimes and also how they studied so they got good grades at school as expressed in the participant quote, ‘when at home you must read so that you can pass and buy a house for your grandparents’.

Personal capability was shown in many ways. The children described school subjects they perceived as easy as facilitators for participating in school, creating structure and capability in their schoolwork, ‘doing work every day instead of letting it pile up’, ‘It is important with education, no hindrance, I encourage myself’. The children further talked about their positive view of themselves to have the capability to be responsible for their own health as a facilitator. Caring for themselves in personal hygiene was also described as a facilitator, ‘clean my body and wash’, ‘to put medicine in the mouth’. A facilitator of looking after their own health was to know how to take medicine with the aid of the father, ‘yogurt or buttermilk helps and dad helps me’.

#### Being included

The children talked of being included and having fun together with others as a facilitator to participation in social activities.

Being included was a facilitator and was described as taking part in conversations and being understood whilst taking care of other family members, ‘getting to sit with family and talk’. A facilitator of participation was also when the child was rewarded for taking care of his or her family members, ‘when keeping up chores and meal preparation, parents take him out to’ or someone prepared birthday parties for the child, ‘they make a birthday party for you’. The children also talked about being included and having fun together with others, ‘to talk with everybody’.

A facilitator of being included at family mealtimes was to be able to participate in the conversations and thus participate in daily family life, ‘you can speak about how was school and mum and dad’s day at work, plans for the week and holidays’. To get help from parents was a facilitator for being included in family life activities, ‘parents help’. When there were relations free of conflicts, the children were included and that was perceived as a facilitator, ‘when I’m communicating with my friends and not fighting’. Furthermore, a skilled educator who adapted the teaching to the needs of the child was perceived by the children as a facilitator, ‘less pressure and more allowance to complete tasks or homework’ and ‘[*w*]hen my teacher explains everything’.

#### Have resources

To be able to have resources to be driven to church facilitated their participation in religious activities, ‘driven there (to church)’.

## Discussion

Barriers reported by the children were related to personal functioning, social exclusion and lack of resources. The facilitators reported by the children included personal capability, being included, and having resources and satisfaction. Interestingly, these seem to be each other’s opposites. It is important to point out that what is considered by one child as a barrier can be seen as a facilitator by another child. The study results were similar to that of Huus et al. (2021), where social exclusion in different situations was reported as a barrier and being included was reported as a facilitator. One of the examples could be the children’s interactions in relationships, both about their own perceived capability to interact with others and about other people’s interaction with the children.

Barriers seemed to be imposed by the children’s general health and well-being. Children with intellectual disabilities may present with co-morbid conditions, such as motor disabilities, and health conditions, such as epilepsy (Gautam, Bhatia & Rathi 2014). It is, therefore, not surprising that the children also reported difficulties in looking after their own health and the fear of medical procedures as barriers. This barrier to participation in personal functioning was often described by the children themselves as being within the child and the child’s perceived view of self as not capable. They were, however, aware of strategies that helped maintain their health, such as eating the correct food. This can be described as a sense of self, which is one of the concepts in the Family of Participation-Related Constructs where a good sense of self facilitates participation, whilst a less good sense of self decreases it (Imms et al. 2017).

Policies and programmes enforced by the school were reported to be quite overwhelming and unsupportive of children’s needs. The programmes were not flexible and did not easily accommodate the children. Rigid and non-inclusive programmes could be a barrier to participation (Guralnick 2017; King et al. 2013). The children were forced to continue with a programme in a manner that brought about discomfort, that is, it was not adapted to suit their needs (Vosloo 2009). These programmes also included complex instructions and requirements when participating in activities. When developing programmes, the Convention on the Rights of the Child emphasises including children themselves when drafting policies and programmes that involve them (Sandland 2017; United Nations General Assembly 1989). In this way, the children are able to advise on programmes that foster and encourage participation, and their activity competence can be enhanced.

The importance of being at school was rated as a facilitator to participation. The children reported the structured routine offered by the school setting as calming, thereby allowing learning to occur – again, stressing the importance of the children being engaged in school activities, not just being physically present at those activities as important for participation (Imms et al. 2017). It is interesting to note that the children could report on educators or trainers who were not knowledgeable about their disability and, therefore, did not know how to adequately assist them. This hindered participation of children in certain activities. However, the presence of an educator who was skilled in adapting activities for their needs assisted them in being part of the activities or completing school tasks. Education systems and services that support the inclusive education of children with disabilities are a facilitator of the participation of such children, as it ensures not only the availability of inclusive schools but also training of educators so that an adapted curriculum is available.

Negative attitudes of educators and the children’s peers represented a barrier to participation. These included social attitudes, such as social exclusion and bullying. The children faced barriers in being included in social networks and, therefore, were not being treated on an equal basis by their peers and educators. Similar findings are reported by Huus et al. (2021), where children with both physical and intellectual disabilities were found to be excluded from social groups (Huus et al. 2021). Moreover, these children also reported being bullied, resulting in a fear of joining social groups. It is notable that the children most likely to be bullied are those with poor communication and motor skills, such as children with intellectual disabilities (Blake et al. 2012). In this view, educators and interventionists should provide disability desensitisation training to both typically developing children and children with disabilities. Furthermore, training should cover the effects of bullying on other children and strategies to prevent and overcome such incidences.

Caregivers tend not to interact with their children with disabilities during story reading, specifically those with communication disabilities (Morwane, Dada & Bornman 2019). Interestingly, in this study, some children observed a lack of communication by the family members with them. Therefore, opportunities for communication regarding everyday routines or changes in routine are limited. Also, a similar relationship was reported with their siblings. Interventions should, therefore, target improving participation within family routines by introducing strategies that facilitate communication with children with disabilities. Interventions should also include a focus on building relationships between siblings (Anaby et al. 2013).

Social support in the form of families is the main facilitator of being able to participate in various activities. The family often tends to encourage children to do more than they are comfortable with and, therefore, pushes them out of their comfort zone. The children perceived their parents as a key facilitator as they felt they are the ones who assist them with difficult tasks. The environment created by families, such as mealtimes, provides the children with opportunities to share information about their day and make meaningful conversation. It thus appears that mealtimes, where families gather, are where opportunities for participation occur. Similarly, being included in social groups and having fun with their peers are reported as a facilitator of participation. The importance of social inclusion in children with disabilities is widely discussed in the literature (Anaby et al. 2013). Conchar et al. (2016) found that children with disabilities perceived having fun with friends as a facilitator of participation. The children reported that they gained a sense of belonging, and felt supported and cared for when part of a social group.

Being in a church provides an environment where the children feel accepted and included. Given that children also reported that being able to go to church was a facilitator for participation, intervention programmes should also consider embedding activities linked to religious activities, particularly for children from countries where religion is more prevalent (Mugeere et al. 2020).

The unavailability of environmental services and systems, such as transportation, restricted participation of children in everyday activities. The children did not have a transport system that allowed ease of access to schools, and therefore, had to walk long distances. Financial constraints restricted their participation in various activities as children were not able to attend the activities they preferred. This has clinical implications as intervention programmes should ensure that activities are accessible to children in both high-and low-resourced contexts, and that they can participate in their preferred activities.

## Conclusion

This study provides much needed data on self-reported information regarding children’s participation, particularly those who present with intellectual disabilities. Taking into consideration the voices of the children with regard to their needs could facilitate participation. Identified barriers and facilitators were mainly related to how other people influence the children on their participation such as their family and educators. This information is important to be considered when developing interventions aimed at enhancing the participation of the children with intellectual disabilities. Furthermore, in the intervention programmes for the children with intellectual disabilities, it is important to address the attitudes of the people surrounding the children to enable participation in activities in both their family and educational and community settings. It is also important to create interventions aiming at enabling children to become self-advocates and to enhance their self-esteem. This study further provides an understanding of what hinders and facilitates the participation of children with disabilities in everyday life activities, as reported by the children themselves. Again, this information is important to consider when planning and designing programmes that facilitate children’s participation at all levels of society.

## Limitations

Although valuable data were obtained from the findings of the study, a number of limitations are noted. Firstly, the study included children from an upper-middle-income country (South Africa) and from a high-income country (Sweden). The differences between the two countries in terms of the availability of systems and services, such as rehabilitation services and inclusive schools, functioning between the children could be varied and therefore barriers experienced are different. Nevertheless, seeing that participation was measured in activities that were important to the children, it can be assumed that these activities are those that they engaged with. Also, this study only included children with intellectual disabilities from urban areas in both countries and, therefore, did not provide a representation of children from more rural settings where it is known that participation in activities is limited.

Secondly, it was observed that the children had at times found it difficult to understand the questions, even though the interviews were performed with the visual aid Talking Mats^MT.^ Some of the children also had difficulty in expressing themselves. To counter this, the interviewers rephrased the questions and gave the children time to answer and to rest in between the questions.

Finally, the sample size was small and was, therefore, not representative of the population of children with intellectual disabilities. Given that little research has been conducted where the voice of children with a disability is heard, future research should consider including a larger sample where children with disabilities are heard.
